# ‘Let Me Know If There’s Anything I Can Do for You’, the Development of a Mobile Application for Adolescents and Young Adults (AYAs) with Cancer and Their Loved Ones to Reconnect after Diagnosis

**DOI:** 10.3390/cancers14051178

**Published:** 2022-02-24

**Authors:** Sophia H. E. Sleeman, Milou J. P. Reuvers, Eveliene Manten-Horst, Bram Verhees, Pandora Patterson, Silvie H. M. Janssen, Olga Husson

**Affiliations:** 1 Dutch AYA ‘Young & Cancer’ Care Network, 3511 DT Utrecht, The Netherlands; sophia@ayazorgnetwerk.nl (S.H.E.S.); e.manten-ayanationaal@iknl.nl (E.M.-H.); bram@hoestie.nl (B.V.); 2 Department of Medical Oncology, Netherlands Cancer Institute, 1066 CX Amsterdam, The Netherlands; m.reuvers@nki.nl (M.J.P.R.); sh.janssen@nki.nl (S.H.M.J.); 3 Hoestie Foundation, 5616 JX Eindhoven, The Netherlands; 4 Research, Evaluation and Policy Unit, Canteen Australia, Sydney, NSW 2042, Australia; pandora.patterson@canteen.org.au; 5 Faculty of Medicine and Health, The University of Sydney, Sydney, NSW 2050, Australia; 6 Department of Psychosocial Research and Epidemiology, Netherlands Cancer Institute, 1066 CX Amsterdam, The Netherlands; 7 Division of Clinical Studies, Institute of Cancer Research, London SM2 5NG, UK

**Keywords:** adolescent and young adult oncology, social support, communication, health related quality of life, digital intervention, E-health

## Abstract

**Simple Summary:**

Previous research describes the issues AYA cancer patients may face when it comes to maintaining social relationships after their diagnosis. Related issues included mutual misconceptions and a lack of understanding of the impact of cancer. The Dutch AYA ‘Young & Cancer’ Care Network co-created the mobile application ‘AYA Match’ to provide support on this matter. Co-creation, in which the target population is directly involved, appears to be an effective way to establish an intervention that applies to their needs. The aim of this study was to describe the cocreational process, characteristics of AYA Match users and their expectations of the app. The application could be useful for a wider audience in the future, such as older cancer patients or individuals dealing with other diseases.

**Abstract:**

Adolescent and young adult (AYA) cancer patients report a need for support to stay in contact with loved ones after diagnosis. In response to this the Dutch AYA ‘Young & Cancer’ Care Network co-created the mobile application ‘AYA Match’. This study describes the cocreational process, the characteristics of the users and their expectations regarding the app. 121 AYA cancer patients and 37 loved ones completed a questionnaire. 68.6% of the loved ones reported ‘staying in contact’ and ‘finding out about the needs and wishes of ‘their AYA’ during this time’ as the main reasons for downloading the application. 41.1% of the AYA cancer patients expected the app to help them communicate to their loved ones what they do or don’t want and need. 60% of the loved ones indicated that they would like to use the application to offer help to ‘their AYA’ with their daily tasks. Patients and their loved ones have similar expectations when it comes to ‘normalizing’ contact, increasing empathy and mutual understanding about needs and emotions. The AYA Match app could be an adequate answer to the issues experienced regarding contact, support and mutual understanding.

## 1. Introduction

In the Netherlands, each year approximately 3900 adolescents and young adults (AYAs), aged 18 to 39 years, are diagnosed with cancer for the first time [1]. They constitute a specific patient group in oncology, considering their unique medical and psychosocial characteristics. AYAs with cancer differ from children and older adults with regard to the broad spectrum of cancer diagnoses, tumor biology and physiology, pharmacology and genetic variance with respect to cancer susceptibility and treatment [2]. Even though AYAs share many cancer-related challenges with pediatric and older adult cancer patients, including short-term (such as hair loss, pain) and long-term effects of treatment (such as fatigue, cognitive problems, increased risk of cardiovascular diseases), these issues may have a larger impact and different effects on AYA cancer patients. Adolescence and young adulthood are challenging life phases of physical, emotional, cognitive and social maturation. Dealing with a life-threatening disease whilst trying to achieve developmental milestones can have a negative impact on the health-related quality of life of AYA cancer patients [3,4,5].

Adolescents and young adults are dealing with important and future-shaping matters such as completing education, becoming financially and socially independent, developing their own identity [4,5], exploring sexuality, building long-term relationships and deciding on whether they want to become a parent [3,5,6]. The sudden disruption of their (social) life, possible changes in physical appearance, confrontation with mortality, increased temporary dependence on informal caregivers like partner or parents, the risk of losing reproductive capacity and other health-related concerns caused by their cancer diagnosis or treatment disturb or even disrupt the ‘normal’ development of these young people [5,6,7,8]. AYAs diagnosed with cancer report lower social functioning levels compared to their healthy peers even two years after diagnosis [8]. They also report more social functioning problems compared with older cancer patients [9,10]. A qualitative study by Breuer and colleagues (2017) stated that AYA cancer patients indicate their family, friends and partners as the most important people to receive social support from [11]. Breuer and colleagues indicate that, besides helpful support, AYA cancer patients also experience negative support, such as people being overly careful instead of treating them ‘normal’ or understating the severity of their illness. They frequently report difficulties when it comes to establishing and maintaining social relationships, due to long-term effects of treatment or having the feeling of being different from their healthy peers [8]. This may result in friends decreasing or even completely breaking off contact [11].

The vast majority of adolescents and young adults are frequent users of digital applications. In 2018, 97% of the Dutch population had access to the internet and 90% of those aged 16 to 74 used the internet daily [12]. This increases the relevance of implementing digital interventions. A study by Allison and colleagues (2021) indicates the value of online platforms where individuals with cancer are provided with the opportunity to discuss experiences, show emotions and feel understood [13].

Dutch AYA cancer patients in particular highly value the use of online communities focused on peer support, when it comes to recognition and receiving advice and support regarding problems. In a study by Kaal and colleagues (2017) on the usefulness of the Dutch online community for AYA cancer patients, almost half of the participants reported feeling less lonely due to being part of an online peer community [14].

Despite the growing evidence of the usefulness of e-health interventions facilitating social and peer support, not many of the available interventions focus on communication and ‘normalizing’ contact with friends and family during illness. Most digital interventions targeting AYAs with cancer, e.g., web-based or mobile applications, focus mainly on physical health and self-management of side and late effects [15].

In 2016, a group of Dutch AYA cancer patients and their loved ones (e.g., friends, family, colleagues) actively put forward the need for support to stay in contact with each other after the cancer diagnosis (www.ayazorgnetwerk.nl, accessed on 27 October 2021). Related issues included mutual misconceptions and the lack of understanding of the impact of cancer, as well as trouble maintaining social relationships. However, the main issue seemed to be that all conversations between AYAs and their loved ones came back to the illness of the AYA. ‘Let me know if there’s anything I can do for you’, often remains unanswered and therefore turns out to be more dismantling than helpful in the relationship. This led to disconnecting AYAs from who they are and what matters to them as a person, beyond their cancer diagnosis. The call of AYAs and their loved ones to the Dutch AYA ‘Young and Cancer’ Care Network, regarding the need for support, led to the co-creational process of developing a digital intervention: the AYA Match app [16]. The process started with an extensive exploratory study conducted by Engelen (2017) assigned to the Dutch Federation of Cancer Patients Organizations examining the needs of AYA patients and their loved ones regarding social contact and mutual understanding after a cancer diagnosis [16].

This article describes (1) the co-creational process of the development of the AYA Match app and its features; (2) the characteristics of the AYA Match app users, and (3) users’ expectations of the AYA Match app.

## 2. Materials and Methods

### 2.1. Co-Creational Process of Application Development

Through the entire co-creational process of developing the ‘AYA Match’ app, the most important precondition was the involvement of the target users: AYA cancer patients and their loved ones. “Co-creation is a process known as bringing different parties together, which produces a blend of ideas leading to a mutually valued outcome” [17].

**Figure 1:**
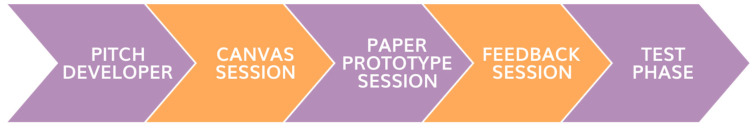
Developmental stages of the co-creational process.

Within the co-creational process, a pitch meeting, a ‘canvas session’, a ‘paper prototype session’, a ‘feedback session’ and a ‘test phase’ were conducted (Figure 1). All sessions and phases were facilitated by the digital developers of the application.

In all phases different AYA cancer patients and/or loved ones were actively involved and considered primary stakeholders throughout the co-creational process. The majority of them had been active within different project groups and think tanks of the Dutch AYA ‘Young & Cancer’ Care Network for a longer period and were therefore considered ‘experts by experience’. Their variety in diagnosis, age, sex and educational level, as well as including new AYAs cancer patients and loved ones during each phase of the cocreational process, contributed to the overall representability.

#### 2.1.1. Pitch Meeting

Five digital development companies were approached by the Dutch AYA ‘Young & Cancer’ Care Network to share their ideas on a digital tool (serious game, mobile application or other). This tool had to meet the needs of AYAs with cancer and their loved ones regarding social support. The tool had to be easy to use and accessible. The ultimate goal was that the AYA cancer patient felt more connected to and understood by friends, family and other acquaintances. The companies pitched their idea in person to several AYA cancer patients and representatives of the Dutch AYA ‘Young & Cancer’ Care Network.

The winning pitch presented the idea of a mobile application called ‘AYA Match’. This idea was chosen by the attending AYA cancer patients and their loved ones. The app was developed by using an iterative approach, meaning repeated testing of a concept by the end users, resulting in an application entirely built around their wishes and needs.

#### 2.1.2. Canvas Session

During the canvas session two AYA cancer patients and a loved one were motivated to discuss and brainstorm through a two hour face-to-face focus group meeting, in order to ascertain which components were needed within the application to reach the desired outcome.

#### 2.1.3. Paper Prototype Session

**Figure 2:**
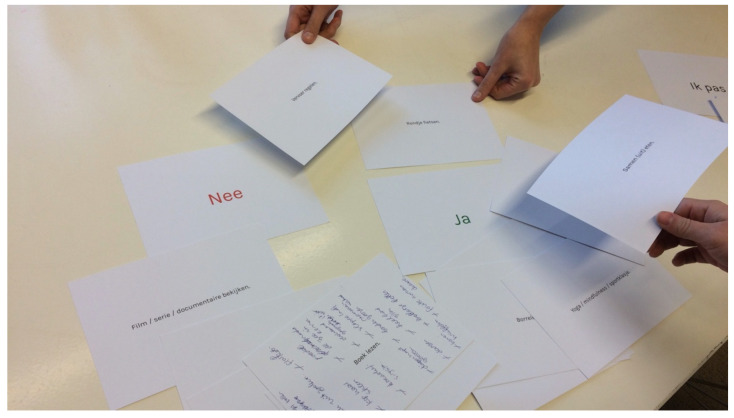
During the paper prototype session AYA cancer patients and their loved ones were invited to interactively try out the concept.

For the paper prototype session seven AYA cancer patients and their loved ones were invited to interactively try out the concept during a face-to-face focus group meeting, using printed cards containing the different screens of the potential application (Figure 2). By actually trying out the concept instead of just presenting it, honest and specific feedback was elicited. During this session oral feedback was obtained on the first nondigital version of the prototype.

#### 2.1.4. Feedback Session

During the feedback session the virtual design of the application was presented on a big screen to five AYA cancer patients and their loved ones, who were able to orally provide the designers with feedback on the visual elements before the start of the *test phase*.

#### 2.1.5. Test Phase

This latter period covered six weeks of testing the digital prototype of the application by five AYA cancer patients and their loved ones. The testing took place at their own homes in their own time. The goal was to find out whether the application stimulated contact between both parties as was aimed for. During this phase, they shared their thoughts on and findings of the different features and screens with the designers via WhatsApp (https://www.whatsapp.com, accessed on 4 February 2017) and an online survey via SurveyMonkey (https://www.surveymonkey.com, accessed on 4 February 2017). The online survey consisted of 13 questions on the usability of the app (such as: ‘What do you find best and worst about the app?’; ‘Is it easy to invite loved ones?’; ‘Are the different features user friendly?’), possible improvement (such as: ‘What changes would you suggest should be made?’; ‘Is the content suitable?’) and whether it served its initial purpose (such as: ‘Do you think the app initiates and stimulates contact?’; ‘Does the app help to discuss heavy topics?’; ‘Does the app cause activities to happen?’).

### 2.2. Characteristics of Application Users and Their Expectations

Once the final concept (titled: AYA Match) was launched as an openly accessible and free mobile application, all users (AYA cancer patients and their loved ones) were invited to participate in a descriptive quantitative questionnaire study.

#### 2.2.1. Eligibility Criteria

Study eligibility criteria included AYAs diagnosed with any type of cancer for the first time between the ages of 18 and 39 years or being a loved one (no age limit) of an AYA cancer patient, including parents, partners, friends and colleagues for example. Participants had to be able to read and understand Dutch.

#### 2.2.2. Procedure

No targeted recruitment regarding the questionnaire study was conducted. All users of the AYA Match app were considered potential participants and invited to take part in the study via the onboarding screens of the app. The app was promoted among different AYA stakeholders in a number of ways. First, the AYA Match app was launched at the Dutch annual AYA ‘Young & Cancer’ congress in March 2018, where attending AYAs were encouraged to download the app. In addition, promotion material, such as flyers, social media posts and a video, was disseminated through patient organizations and health care professionals during the following months. Everyone who downloaded the app was invited to take part in the study and to complete the baseline questionnaire online before they started using the app. If a user decided not to take part in the study, there were no consequences for their further usage of the app. Those who decided to participate were automatically forwarded to the online informed consent form and baseline questionnaire. Two versions of the baseline questionnaire were created: one for AYA cancer patients and one for their loved ones.

Questionnaires were programmed within the online survey platform ‘SurveyMonkey’.

#### 2.2.3. AYA Cancer Patient Survey

The questionnaire for the AYA cancer patients consisted of, among others, questions on sociodemographic and clinical data, an item on their intrinsic motivation to download the app and items on their expectations of usage (such as ‘Who (e.g., family, friends, colleagues) would you like to invite to use the app with?’; ‘What are you hoping the app will help you with?’).

#### 2.2.4. Loved One Survey

The questionnaire for the loved ones consisted of, among others, questions on sociodemographic data, clinical data of ‘their AYA’, an item on their intrinsic motivation to download the app and an item on their expectations of usage (such as: ‘What is the reason you downloaded the app?’, ‘What are you hoping the app will help you and ‘your AYA’ with?’).

#### 2.2.5. Data Analysis

Analyses were performed using SPSS statistical software (version 26, Chicago, IL, USA). Descriptive statistics and frequencies concerning sociodemographic data, clinical data, and user expectations were calculated.

## 3. Results

### 3.1. Co-Creational Process of Application Development

#### 3.1.1. Pitch Meeting

The winning pitch presented the idea of a mobile application offering support on staying in contact after a cancer diagnosis: AYA Match.

#### 3.1.2. Canvas Session

During the canvas session, the purpose of the application was defined and described as follows:

*‘Create a tool to spark contact between both parties:*

*The app (re)connects AYA cancer patients with their loved ones (e.g., family, friends, colleagues). In a casual, unforced way.’*

*Questions that were difficult to ask become less heavy. ‘Let me know if there’s anything I can do for you’ will no longer remain unanswered.*

*An ‘appetizer’ to spark contact, fitting the moment.*

Feedback obtained during the canvas session included comments from loved ones such as:

*“Who am I? Is it up to me to bring up this topic? I have no idea if they have someone to talk to?”, “I’m not sure what they want or don’t want. I find it complicated to ask. I’m not sure if it’s awkward to ask about their needs…” and “I would love to help, but I’m not sure how to do that. I said: let me know if there’s anything I can do, but haven’t heard from them since…”.*

This feedback was used by the digital developers to create a paper prototype to try out and discuss with the AYA cancer patients and their loved ones in the next phase.

#### 3.1.3. Paper Prototype Session

The paper prototype was used to try out and prioritize possible features within the app, resulting in the further development of the two key elements:

1. ‘Rules to play’ is meant to inform loved ones on the patient’s preferences regarding communication around their illness. The patient can ‘like’, ‘dislike’ or ‘pass’ on the different statements. The loved ones can view the patient’s preferences;
2. The ‘activity cards’ give both parties the opportunity to match on a variety of fun things to do, and to ask for (patient) or offer (loved one) help. The activity cards are followed up by a feature where the patient can choose a match with the person of preference and continue to set a date in the built-in chat feature.

These two elements mainly focus on the practical aspect of maintaining contact: ‘how can I ask for/offer help’, ‘what are topics the patient does or does not want to talk about’ and ‘how can I communicate my needs’. More game-oriented elements were suggested, but not preferred by the involved AYA cancer patients and loved ones.

Feedback obtained during the paper prototype session included comments such as: “if I would’ve had access to an app like this during my treatment, I am absolutely sure I wouldn’t have lost as many friends as I did”, confirming the usefulness of the concept. In addition, ideas for refinement were listed, such as “we need to add more humor”.

#### 3.1.4. Feedback Session

**Figure 3:**
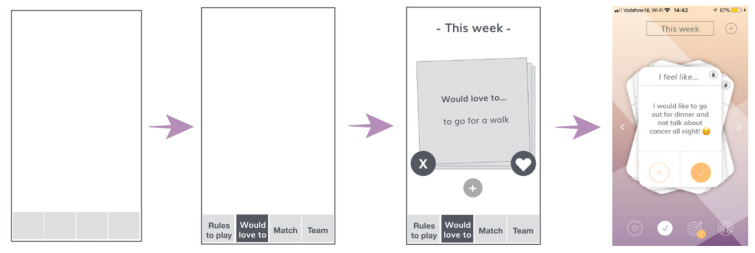
Flowchart of the different phases of the development of the application.

The feedback session was used to present the concept of the different screens and features of the first prototype of the AYA Match app. The visual aspect of the app was developed and presented step-by-step, as shown in Figure 3. The visual elements (e.g., colors, typography, navigation bar and buttons) were designed and adjusted based on the preferences of the patients and loved ones present.

#### 3.1.5. Test Phase

During the six-week test phase AYA patients and their loved ones were asked to test the prototype version of the application. The application developer IJsfontein obtained their final feedback through SurveyMonkey and WhatsApp.

The reports of the testers included positive feedback on easy usage (quote 1, 12, 15) and efficiency (quote 5, 7, 9, 10, 12, 13, 14, 18) (Table 1).

Based on the reported outcomes, final adjustments were made such as the option to add a profile picture (quote 3), structured onboarding (quote 2, 6, 8), a built-in chat function (quote 4), the possibility to skip propositions on certain topics (quote 9, 11), creating an overview of matches per person (quote 16) and activating push notifications to increase engagement (quote 17).

#### 3.1.6. The Launch of the Application

**Figure 4:**
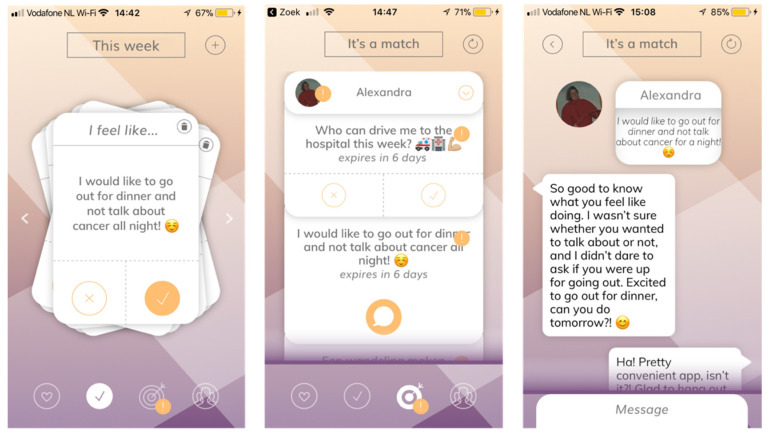
Different screens of the final product: AYA Match. More is available in this demo video in the Supplementary Materials (Video S1).

The final phase was kicked off with the official launch of the application, which enabled anyone interested to download and start using AYA Match. The application ended up with two main features, as described in Section 3.1.2 and shown in Figure 4.

### 3.2. Characteristics of AYA Match App Users

Participants were not personally invited and as a result no detailed information of respondents versus non-respondents was available.

#### 3.2.1. AYA Cancer Patients

In total 141 participants took part in the study. However, three did not complete the full questionnaire and 17 were outside the AYA age range at time of diagnosis, resulting in 121 eligible participants. Completing the questionnaire took approximately eleven minutes. Table 2 displays the sociodemographic and clinical characteristics of the participants. Among them were 100 female and 21 male participants. Most of the participants (*n* = 114) were residents of the Netherlands, versus seven Belgian participants. Mean age at baseline was 28.8 ± 4.3 years old. Mean age at diagnosis was 27.2 ± 4.3 years old. Thirty-eight percent (*n* = 46) of participants were diagnosed with breast cancer. Forty-nine participants (40.5%) were within their first year after diagnosis, 52.1% were between one and five years after their diagnosis (*n* = 63) and nine were between six to ten years post diagnosis.

#### 3.2.2. Loved Ones of AYA Cancer Patients

In total, 74 loved ones participated in the study. However, seven of the participants completed the baseline questionnaire twice, resulting in a total of 67 participants. Of them, 30 were excluded because the patient who invited them to use the app was outside the AYA age range (*n* = 37). Completing the questionnaire took approximately nine minutes. Sociodemographic and clinical data are reported in Table 2.

Among the eligible participants were 27 females and 10 males. 33 participants were residents of the Netherlands, versus 4 Belgian participants. Mean age at baseline was 39.9 ± 14.7 years old. Almost half of them (45.9%) were friends, others were partners, family members and colleagues. Fifty-one percent of ‘their AYAs’ were on active treatment at baseline.

### 3.3. Expectations of AYA Match App Users

Table 3 describes the expectations of the remaining participants (*n* = 107, AYA cancer patients, *n* = 35 loved ones) of the AYA Match app. Almost 25 percent of patients reported ‘staying in contact’ as an important reason for downloading the application, as did 68.6% of the loved ones.

60.0% of the loved ones indicated that they would like to use the application to offer help to their AYA with their daily tasks. 71.0% of the patients would like to use the application with their close friends, but they also reported considering inviting their parents (41.1%) and their partner (35.5%).

## 4. Discussion

The primary aim of this study was to describe the co-creational process of developing the AYA Match app and report the characteristics of its users and their expectations of the app. Over 40% of AYA cancer patients included in this study indicated the hope that using AYA Match would help them clarify to loved ones what they want and need. Over 65% of responding loved ones stated they expected the app would help them understand what they could do to support ‘their AYA’. In addition, 43% reported the app might help them gain a better understanding of the situation ‘their AYA’ was in and how they are feeling. This study has shown that AYA cancer patients and loved ones have similar expectations of the app when it comes to contact and mutual understanding after a cancer diagnosis. Due to the process of co-creation, the needs of both parties have been integrated into a digital intervention. As far as known to the authors, there aren’t many comparable apps described in literature. AYA Match intervenes on communication with friends and family during treatment, while most digital interventions focus on lifestyle, symptom registration, communication with doctors and other cancer patients [15].

It is notable that the vast majority of respondents in this study were female, which is likely related to the high number of breast cancer patients. Most of the included AYA cancer patients were between 18–29 years of age at diagnosis, and over half of the participants were in the phase of active treatment. This indicates that AYAs in active treatment might experience a bigger need for support in communication and contact with the people around them compared to those who already completed treatment. Almost all AYA cancer patients and loved ones finished higher level education. A study by Jansen and colleagues (2015) demonstrates that being younger, higher educated and under active treatment is related to a positive outlook on the use of eHealth [18]. Caroll and colleagues (2017) reported that patients of older age, male gender and who have a lower educational level (secondary vocational education or lower) indicated a reduced probability of using a health app [19].

### 4.1. Value of Co-Creation

Co-creation can be seen as an important aspect of a participatory design (PD). This is a collective and creative process, which includes five phases: exploration, creation, testing, implementing and refreshing. “In PD, one of the guiding principles is that every relevant stakeholder can and should be involved in the design process.” The Dutch AYA ‘Young & Cancer’ Care Network developed a method for successfully involving AYA cancer patients in improving age-specific healthcare and tools, such as the AYA Match app [20].

A study by Bjerkan and colleagues (2014) endorses the value of a flat structure, a method in which patients’ expectations and their knowledge is directly communicated to system developers. The flat structure was used while creating the AYA Match app. By creating a confidential environment wherein all stakeholders are represented, individual needs, expectations and new ideas are expressed and used to the utmost potential [21].

This indicates that co-creation can be a useful method of converting the patients’ needs and wishes into a self-management intervention. The similarity between the input of patients and loved ones collected during the co-creational process and the expectations of the actual users of the intervention, underline the usefulness of co-creation in the development of the AYA Match app.

A study by McNeil (2019) indicates that social support from family and peers is of significant importance for AYA cancer patients. This support, however, is a changing concept over time and may thus differ throughout one’s treatment period [22]. Breuer and colleagues (2019) reported that the relationships with loved ones are affecting how the AYA cancer patient feels, by providing support and listening to them. Undertaking activities together, as the AYA Match app stimulates, was experienced as distracting from living with a disease and perceived as pleasant. The participants in the study indicated that it was helpful if loved ones were available and reachable throughout treatment [11]. These studies substantiate the importance of the AYA Match app. By indicating one’s needs for support at individual moments in time, this application responds to the changing aspect of support and facilitates continuous accessible contact with loved ones.

It should be taken into account that the co-creational process described in this paper was conducted in a small sample of AYA cancer patients and their loved ones. In addition, informed consent was not sought during the co-creational process. As a result it was not possible to share their sociodemographical data. This may make generalization more difficult. It could be challenging to recruit many diverse participants, as was experienced during recruitment. However, research has shown that it is not necessary to have a large group in order to properly implement a participatory design [20]. The AYAs and loved ones who contributed in the co-creational process have been involved in their patient organization for some time and are thus familiar with the needs of other patients and their loved ones, besides their own experiences. A larger group may be recruited for the follow-up research, but for this developmental study, the contribution of these participants was estimated to be very valuable.

During the developmental phase of the AYA Match app, the AYAs involved in the co-creational process were almost all in the post-treatment phase. The problem of recruiting patients undergoing active treatment is widely acknowledged in literature but under-researched [23,24]. In addition to factors identified by Van den Brink et al. (2020) such as low disease incidence, low project awareness, and high patient burdens (e.g., time commitment), we propose additional factors specific for our target group of AYAs, namely (1) low physiological and/or emotional wellbeing as a result of the disease and/or treatment, (2) high (perceived) risks of meeting up in groups (e.g., due to compromised immune systems), and (3) high avoidance of emotional distress (e.g., reflecting on the social-psychological impact of one’s disease) [24]. While a deep dive into these factors was out of scope for our project, this stands as a key recommendation for further research.

Our survey has been carried out on a very diverse population, given the broad age range and cancer types of the participating AYAs. This increases the external validity of the research findings and allows for possible generalization. Yet generalizing the results of this study should be done with caution, given its limitations. Those who participated in this research included a large number of female cancer patients and many patients were in active treatment at the time of downloading the application. The need to improve social support and contact with friends and family is not only important during treatment: patients are in need of interventions to decrease the lack of social support post-treatment as well. Research indicates a significant drop in the quantity of social support from loved ones over the first-year post-treatment [25]. In addition, it has been found that AYA cancer patients in particular score significantly lower on social functioning up to two years after diagnosis compared to their healthy peers, and one-third of them remain at risk of poor social functioning [8]. Therefore, an intervention that helps harmonizing the social relationships of the AYA during treatment might prevent social isolation later on.

This study was conducted using self-reporting, which is limited by recall bias. Also, self-reports have a risk of social-desirable answers. It could be difficult to admit that one wished for more social support or that one does not want it to be inferior to the support they did receive. As the questionnaire focused on user expectations and therefore was completed once, the answers might be influenced by one’s mood at that time.

### 4.2. Next Phase

This study adds to the current knowledge by identifying the needs and wishes of AYA cancer patients regarding social support and their outlook on using applications to help them receive this. Social support coming from friends and family during times of treatment and rehabilitation contribute to the extent of how well patients adjust to their new situation [26]. However, staying in contact and leveling with family and friends can be difficult after a cancer diagnosis. Especially young cancer patients are at risk of poor social functioning and therefore in need of interventions stimulating social support [8]. A review by Tremolada and colleagues (2016) demonstrates that female AYA cancer patients prefer to ask directly for support from family and friends when it comes to solving problems. Male AYA cancer patients favor more indirect strategies to receive support, for example initiating pleasant, physical activities [27]. In addition, even though the application has been developed in co-creation, the design may not be attractive to men. Future research should be conducted to determine potential gender differences in use of the application that could inform future refinement of the application to make it attractive for both sexes.

The AYA Match app could potentially be considered an adequate answer to the needs of AYA cancer patients and their loved ones, as it focuses on initiating and maintaining contact. Both the possibility to seek support among loved ones and the encouragement of undertaking activities together are facilitated within the intervention, serving both the preferences of males and females as described by Tremolada [27]. Therefore, extended research is being conducted to measure the effect of using AYA Match on social functioning and perceived social support during treatment, as well as the impact of a cancer diagnosis on the social life of AYA patients.

Parallel to the continuous research, partnerships with fellow organizations are established to create sustainable support for further development of the application and make it available to a larger number of people. Several parties have shown their interest in the AYA Match app, including healthcare providers of pediatric- and older adult cancer patients, as well as (international) patient organizations of other diseases and humanitarian organizations. This interest has been endorsed by the 30 loved ones who were using the app, but were excluded in this study due to ‘their AYA’ being outside the AYA age range.

In extend to the search for sustainable support, the AYA Match app is being re-explored and possibly renamed to make it more appealing to patients outside the AYA age range.

## 5. Conclusions

In conclusion, the results as described in this study demonstrate that the application could be an adequate answer to the issues AYA cancer patients and their loved ones experience when it comes to contact and mutual understanding after a cancer diagnosis. Co-creation, in which the target population is directly involved in the process of developing an intervention, appears to be an effective way to establish an intervention which applies to their needs. By sharing our knowledge and possibly including other patient populations, such as older cancer patients or individuals dealing with other diseases, the application may become available for a wider audience in the future.

## Figures and Tables

**Table 1:** Quotes of AYAs and loved ones during the six-week test phase.

Domain/Feature	Nr. Quote	Quote ^1^
Overall usage	1	“The app has surprised me! It’s very basic and easy to use, which makes it very accessible.”
	2	“Maybe add a button to go back to the onboarding instructions.”
	3	“It would be nice to add a profile picture.’’
	4	“Once you have a match, it’d be convenient if there’s a built-in chat function to plan the activity right away.”
	5	“By using the application, you no longer need to ask over and over again asking whether there’s anything you can do to help, which can become quite annoying for both parties.”
	6	“I would like an introduction to the app: what’s the purpose, how does it work?”
	7	“To me the best part of the app is the lightness to discuss heavy topics and the way small, yet important moments of social contact get initiated.”
Onboarding	8	“I’d advise starting the app with the screen where you can add friends. It took me a couple of minutes to figure out where to start. (..) Using a unique code to add people to my team feels safe to me.”
Rules to Play	9	“The app makes it easier to talk about difficult topics. Some of the questions are not yet applicable, such as the ones about death, but are already between the options. I had a hard time with those.”
	10	“The funny cards are helpful to initiate talking about difficult topics.”
	11	“I thought the propositions were too limited. You should be able to pick them yourself. If chances of survival are high, you might not want to receive questions on death. This is not applicable at all times. It would be nice to be able to delete those.’’
	12	“Clear propositions, nice design with the little cards. Very easy to choose between ‘yes’ or ‘no’. (...) Gives useful insight for me as a friend on how to help ‘my AYA’.”
Activities	13	“It’s nice that the application gives the opportunity to display your needs through cards. And it’s clever the other person doesn’t get notified of a rejection when I decide I don’t want to match on a certain activity.’’
	14	“I like the activity cards a lot! Due to the app I listened to my guilty music pleasures for the first time in a while. And I even had a ‘date’ with my boyfriend, by making time to watch a movie together. The app brought me new ideas.”
Match	15	“The app gives insight into if one would like to do an activity. You only need to send a text and the date is set.”
	16	“Once you’ve got matches with multiple people, the overview gets a bit lost. Clustering matches per person would be helpful.”
	17	“I would like push notifications and new topics to discuss as an impulse to use the app again.”
	18	“I liked being brought on with ideas to meet up. It gets you out of your comfort zone or routine.”

^1^ All quotes in this table are translated from Dutch to English.

**Table 2:** Sociodemographic and clinical data of the Adolescent and Young Adult (AYA) cancer patients and loved ones using the AYA Match app.

Characteristic	AYAs	Loved Ones
	*n* (%)	*n* (%)
Sex		
Female	100 (82.6)	27 (73.0)
Male	21 (17.4)	10 (27.0)
Marital status		
Married or living with partner	62 (50.4)	25 (67.6)
In a relationship, not living together	10 (8.2)	3 (8.1)
Divorced	1 (0.8)	2 (5.4)
Single	48 (39.6)	7 (18.9)
Children		
Yes	30 (24.8)	n/a ^1^
No	91 (75.2)	n/a
Living situation		
With (foster) parents	28 (23.1)	n/a
Living alone	20 (16.5)	n/a
Living with housemates	7 (5.8)	n/a
Living with partner/children	60 (49.6)	n/a
Other	6 (5.0)	n/a
Educational level		
Secondary vocational education	10 (8.2)	4 (10.8)
Higher vocational education	52 (43.0)	11 (29.7)
University	59 (48.8)	22 (59.5)
Age at diagnosis, y		
18–23	21 (17.4)	n/a
24–29	62 (51.2)	n/a
30–39	38 (31.4)	n/a
Years past diagnosis		
Within the first year	49 (40.5)	n/a
1–5 years	63 (52.1)	n/a
6–10 years	9 (7.4)	n/a
Type of cancer		
Breast	46 (38.0)	n/a
Testis	6 (5.0)	n/a
Sarcoma	2 (1.6)	n/a
Leukemia	9 (7.4)	n/a
Hodgkin Lymphoma	14 (11.6)	n/a
Non-Hodgkin Lymphoma	9 (7.4)	n/a
Brain	7 (5.8)	n/a
Melanoma	8 (6.6)	n/a
Cervix	7 (5.8)	n/a
Colorectal	1 (0.8)	n/a
Thyroid	7 (5.8)	n/a
Lung	3 (2.5)	n/a
Other	9 (7.4)	n/a
Phase of treatment (of ‘related AYA’)		
Active	65 (53.7)	19 (51.4)
Wait-and-see	11 (9.1)	4 (10.8)
In remission	31 (25.6)	5 (13.5)
Palliative	6 (5.0)	4 (10.8)
No treatment plan yet	4 (3.3)	3 (8.1)
Other	4 (3.3)	0 (0.0)
Secondary disease interfering with daily functioning ^2^		
None	77 (63.6)	n/a
Physical disease	28 (22.4)	n/a
Psychological disease	18 (14.9)	n/a
Relationship with AYA ^2^		
Parent	n/a	5 (13.5)
Sibling	n/a	3 (8.1)
Partner	n/a	3 (8.1)
Family member	n/a	3 (8.1)
Friend	n/a	17 (45.9)
Colleague	n/a	4 (10.8)
Acquaintance	n/a	3 (8.1)
Other	n/a	4 (10.8)

^1^ n/a = not applicable; ^2^ Participants were allowed to select multiple options, percentages do not add up to 100.

**Table 3:** Expectations of Adolescent and Young Adult (AYA) cancer patients and their loved ones before usage of the AYA Match App.

Variable	AYAs ^1^	Loved Ones ^2^
	*n* (%)	*n* (%)
Reasons for downloading AYA Match, I … ^3^		
… would like to stay in contact with loved ones	25 (23.4)	n/a ^4^
… would like to make contact with loved ones less ‘heavy’	19 (17.8)	n/a
… would like to receive better understanding from loved ones	19 (17.8)	n/a
… would like to share needs and wishes in a neutral way	23 (21.5)	n/a
… was tipped by someone else	19 (17.8)	2 (5.7)
… was curious	82 (76.6)	11 (31.4)
… have another reason ^5^	10 (9.3)	7 (20.0)
… would like to stay in contact with ‘my AYA’	n/a	24 (68.6)
… would like to know the needs and wishes of ‘my AYA’ during this time	n/a	24 (68.6)
… would like to emotionally support ‘my AYA’	n/a	19 (54.3)
… would like to know how I can help ‘my AYA’ out with daily tasks	n/a	21 (60.0)
… would like to have better understanding of what ‘my AYA’ is going through	n/a	15 (42.9)
… would like to prevent a change in contact because of the cancer	n/a	5 (14.3)
I’d like to invite my … to use AYA Match with me ^3^		
Close friends	76 (71.0)	n/a
Friends	12 (11.2)	n/a
Parents	43 (40.2)	n/a
Partner	38 (35.5)	n/a
Family	22 (20.6)	n/a
Household	21 (19.6)	n/a
Class/study mates	5 (4.7)	n/a
Colleagues	17 (15.9)	n/a
Expectations: I hope AYA Match helps … ^3^		
… making contacting me less ‘scary’	32 (29.9)	n/a
… to specify what I do/don’t want and need	44 (41.1)	n/a
… to make it easier to help me	25 (23.4)	n/a
… to receive more understanding from my surroundings	26 (24.3)	n/a
… to diminish the amount of energy to stay in contact with my surroundings	34 (31.8)	n/a
No specific expectations	52 (48.6)	10 (28.6)
… making our contact feel less uncomfortable or ‘forced’	n/a	2 (5.7)
… me understand what may or may not help ‘my AYA’	n/a	23 (65.7)
… me understand the situation ‘my AYA’ is in, and how he/she feels	n/a	15 (42.9)
… to make staying in contact easier	n/a	8 (22.9)

^1^ Among the 121 participants, as described in Table 1, fourteen did not complete the concluding part of the questionnaire about their expectations of the AYA Match app; ^2^ Among the 37 participants, as described in Table 1, two did not complete the concluding part of the questionnaire about their expectations of the AYA Match app; ^3^ Participants were allowed to select multiple options, percentages do not add up to 100; ^4^ n/a = not applicable; ^5^ Additional reasons mentioned by respondents included: ‘I’ll accept all the help I can get’, ‘I’d like to get in touch with other patients’, ‘I’ve been asked to test the application by my patient organization’, ‘It’d be great to talk to someone who understands my situation’, ‘My doctor referred me’, ‘I have trouble asking for help and I hope this app will help’, ‘I’d like to communicate my needs to a group of people at once’, ‘I’d like to stay updated about my loved one with cancer’, ‘I mainly hope this app helps my AYA’.

## Data Availability

The data presented in this study are available on request from the corresponding author.

## Supplementary Materials

The following supporting information can be downloaded at https://youtu.be/78EAqBUxHAM (accessed on 5 December 2018), Video S1: Demo video for AYA Match app.
